# The TRIFLOW study: a randomised, cross-over study evaluating the effects of extrafine beclometasone/formoterol/glycopyrronium on gas trapping in COPD

**DOI:** 10.1186/s12931-020-01589-5

**Published:** 2020-12-09

**Authors:** James Dean, Catalina Panainte, Naimat Khan, Dave Singh

**Affiliations:** 1 Medicines Evaluation Unit, Southmoor Road, Manchester, M23 9QZ UK; 2 Division of Infection, Immunity and Respiratory Medicine, School of Biological Sciences, Faculty of Biology, Medicine and Health, Manchester Academic Health Science Centre, The University of Manchester and Manchester University NHS Foundation Trust, Manchester, UK

**Keywords:** COPD, Triple therapy, Small airways, Oscillometry

## Abstract

**Background:**

The effects of triple therapy on gas trapping in COPD are not fully understood. We evaluated the effects of the long acting bronchodilator components of the extrafine single inhaler triple therapy beclometasone dipropionate/formoterol/glycopyrronium (BDP/F/G) pMDI on gas trapping.

**Methods:**

This open-label, randomised, single centre, 2-way cross-over study recruited 23 COPD patients taking inhaled corticosteroid combination treatments and with residual volume (RV) > 120% predicted at screening. Inhaled BDP was taken during run-in and washout periods. Baseline lung function (spirometry, lung volumes, oscillometry) was measured over 12 h prior to randomisation to BDP/F/G or BDP/F for 5 days followed by washout and crossover. Lung function was measured prior to dosing on day 1 and for 12 h post-dose on day 5.

**Results:**

Co-primary endpoint analysis: BDP/F/G had a greater effect than BDP/F on FEV_1_ area under the curve over 12 h (AUC_0–12_) (mean difference 104 mls, p = 0.0071) and RV AUC_0–12_ (mean difference − 163 mls, p = 0.0028). Oscillometry measurements showed a greater effect of BDP/F/G on the difference between resistance at 5 and 20 Hz (R5–R20) AUC_0–12_, which measures small airway resistance (mean difference − 0.045 kPa/L/s, p = 0.0002). Comparison of BDP/F with the baseline measurements (BDP alone) showed that F increased FEV_1_ AUC_0–12_ (mean difference 227 mls) and improved RV AUC_0–12_ (mean difference − 558 mls) and R5–R20 AUC_0–12_ (mean difference − 0.117 kPa/L/s), all p < 0.0001.

**Conclusions:**

In COPD patients with hyperinflation, the G and F components of extrafine BDP/F/G improved FEV_1_, RV and small airway function. These long acting bronchodilators target small airway function, thereby improving gas trapping and airflow.

*Trial registration* The study was retrospectively registered at ClinicalTrials.gov on 15th February 2019 (No.: NCT03842904, https://clinicaltrials.gov/ct2/show/NCT03842904).

## Background

Chronic obstructive pulmonary disease (COPD) is characterised by persistent inflammation in the small airways, associated with remodelling and ultimately small airway destruction [1, 2]. These pathological changes reduce airflow and increase gas trapping [3]. Inhaled treatment with a long acting beta-agonist (LABA) and/or a long acting muscarinic antagonist (LAMA) can bronchodilate the small airways to improve airflow, reduce air trapping and thereby improve dyspnoea and exercise capacity [4–7]. Inhaled corticosteroid (ICS) treatment can target small airway inflammation to improve clinical outcomes including exacerbation rates [8].

The triple combination inhaler containing beclometasone dipropionate/formoterol/glycopyrronium (BDP/F/G) was developed as an extrafine formulation to enable efficient delivery of an ICS combined with a LABA and a LAMA to the small airways [9]. Phase 3 clinical trials have shown that this single inhaler triple therapy reduces exacerbation rates, and improves quality of life and lung function compared to double combination inhalers containing ICS/LABA or LAMA/LABA, and compared to LAMA monotherapy [10–12].

In order to further understand the effects of the bronchodilator components of extrafine BDP/F/G on the small airways and gas trapping, we conducted a clinical trial focusing on lung volumes and small airway physiology. To measure effects on gas trapping, we recruited patients with increased residual volume (RV) at study entry. The TRIFLOW study was a randomised, cross-over study evaluating the effects of the F and G components of BDP/F/G on lung volumes and small airway physiology (using oscillometry).

## Methods

### Participants

This study recruited male and female COPD patients from 40 to 75 years of age, with a post-bronchodilator forced expired volume in 1 s (FEV_1_) of 30–80% predicted, a FEV_1_/forced vital capacity (FVC) ratio of < 0.70, and a RV > 120% predicted. Eligible patients were current or ex-smokers with a smoking history of ≥ 10 pack years. All patients were required to be currently taking ICS as part of treatment with ICS/LABA or ICS/LABA/LAMA (as separate inhalers or a single inhaler). Patients were excluded using the following criteria: known respiratory disorder other than COPD; COPD exacerbation within 8 weeks or hospitalisation within 12 months due to COPD; or abnormal clinically relevant findings on physical examination, laboratory or electrocardiogram (ECG) evaluations that in the investigator`s opinion made it unsafe for the patient to participate. All patients provided written informed consent using a protocol approved by the Health & Social Care Research Ethics Committee A (18/NI/0194).

### Study design

**Fig. 1:**
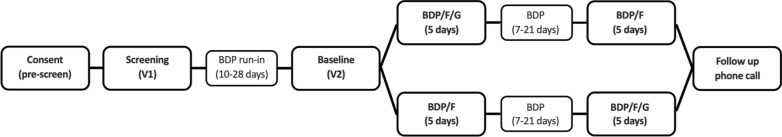
Study design. *BDP* beclometasone dipropionate, *F* formoterol, *G* glycopyrronium

The study was a randomised, open label, 2 way cross over design (ClinicalTrials.gov registration: NCT03842904). Eligible patients commenced a 10–28 day run-in period receiving BDP (Clenil Modulite) 200 µg twice daily in place of current ICS treatment. Long acting bronchodilator treatments were withdrawn, and replaced with short acting bronchodilators for use as needed. After ≥ 10 days of run-in, a baseline visit was completed, followed by two treatment periods of 5 days each, separated by a washout period of 7–21 days during which BDP and short acting bronchodilators were used (Fig. 1). Patients were randomised (with a 1:1 ratio), to receive triple therapy (Trimbow: BDP/F/G, 100/6/10 µg pMDI, 2 puffs, twice daily) followed by dual therapy (Fostair: BDP/F, 100/6 µg pMDI, 2 puffs, twice daily) or vice-versa. BDP/F/G or BDP/F treatment was commenced on the morning of day 1 and a final dose given on the morning of day 5 (total of 9 doses).

At the baseline visit, and on day 5 of each treatment period, impulse oscillometry (IOS), spirometry, and whole body plethysmography were performed (in that order) prior to the morning dose. IOS and spirometry were then repeated 30 min and 1, 2, 4, 6, 8, 10 and 12 h post-dose. Plethysmography was repeated at 1, 2, 4, 8 and 12 h post-dose. On day 1 of each treatment period, patients performed spirometry, IOS, and whole body plethysmography prior to the first administration of BDP/F/G or BDP/F.

### Lung function

IOS was performed on the IOS Masterscreen system (Erich Jaeger, Hoechberg, Germany), to European Respiratory Society (ERS) standards [13]. Acceptable tests had a coherence index of ≥ 0.7 for both 5 and 20 Hz, and the average values reported from three tests where R5, R20 and Fres values were within 10% of their respective mean values. Spirometry was performed on the NDD Easy On-PC system (NDD medical technologies, Zurich, Switzerland), to American Thoracic Society (ATS)/ERS standards [14]. Predicted values were calculated using the global lung function initiative (GLI) 2012 equations [15]. Whole body plethysmography was performed on the Vmax Encore system (CareFusion, Hoechberg, Germany), to ATS/ERS standards [16]. Thoracic gas volume (TGV) was quantified for three acceptable tests, where the associated functional residual capacity (FRC) values were within 5% of the mean value. Vital capacity (VC) manoeuvres were then performed in triplicate to ascertain residual volume (RV) and total lung capacity (TLC), where the highest two VC values agreed within 150 mls. Predicted values were calculated using the ERS 1993 equations [17]. The following measurements were collected: FEV_1_; forced vital capacity (FVC); mid-expiratory flow (FEF25–75%); RV; TLC; FRC; inspiratory capacity (IC); specific airway conductance (sGaw); airway resistance (Raw); peripheral respiratory resistance (R5–R20); expiratory flow limitation (∆X5); total respiratory resistance (R5); reactance (X5); resonance frequency (F*res*); reactance area (AX);

### Statistical methods

The co-primary objectives were to compare the effects of BDP/F/G versus BDP/F on change in FEV_1_ and RV on day 5 compared to day 1 pre-dose, by analysing area under the curve over 12 h (AUC_0–12_). The secondary objectives were to compare the effect of BDP/F/G versus BDP/F on change in all other lung function measurements for AUC_0–12_ and peak, while treatment differences for FEV_1_ and RV peak and trough (12 h post-dose) were also determined. Additionally, the differences between AUC_0–12_ for BDP/F/G and BDP/F at day 5 were compared to the AUC_0–12_ for BDP alone at baseline visit (relative to baseline visit pre-dose value). A sample size of 20 patients was required to detect a mean difference > 0.23 L (FEV_1_ AUC_0–12_) between treatments. This corresponds to a 2-sided t-test with 80% power conducted at the 5% significance level, assuming a standard deviation of 0.17 L for the paired differences between treatment groups. The primary analysis was conducted using a mixed model. The model included fixed effects for treatment and period and a random effect for patient within treatment sequence. For the primary endpoint, the AUC_0–12_ from the baseline visit was included as a covariate. The AUC_0–12_ (normalised by time), peak and trough (defined as the 12 h measurement post-dose on day 5) values were analysed as change from within-period day 1 pre-dose measurement. All were analysed without any procedures to account for multiple comparisons.

## Results

### Participants

**Fig. 2:**
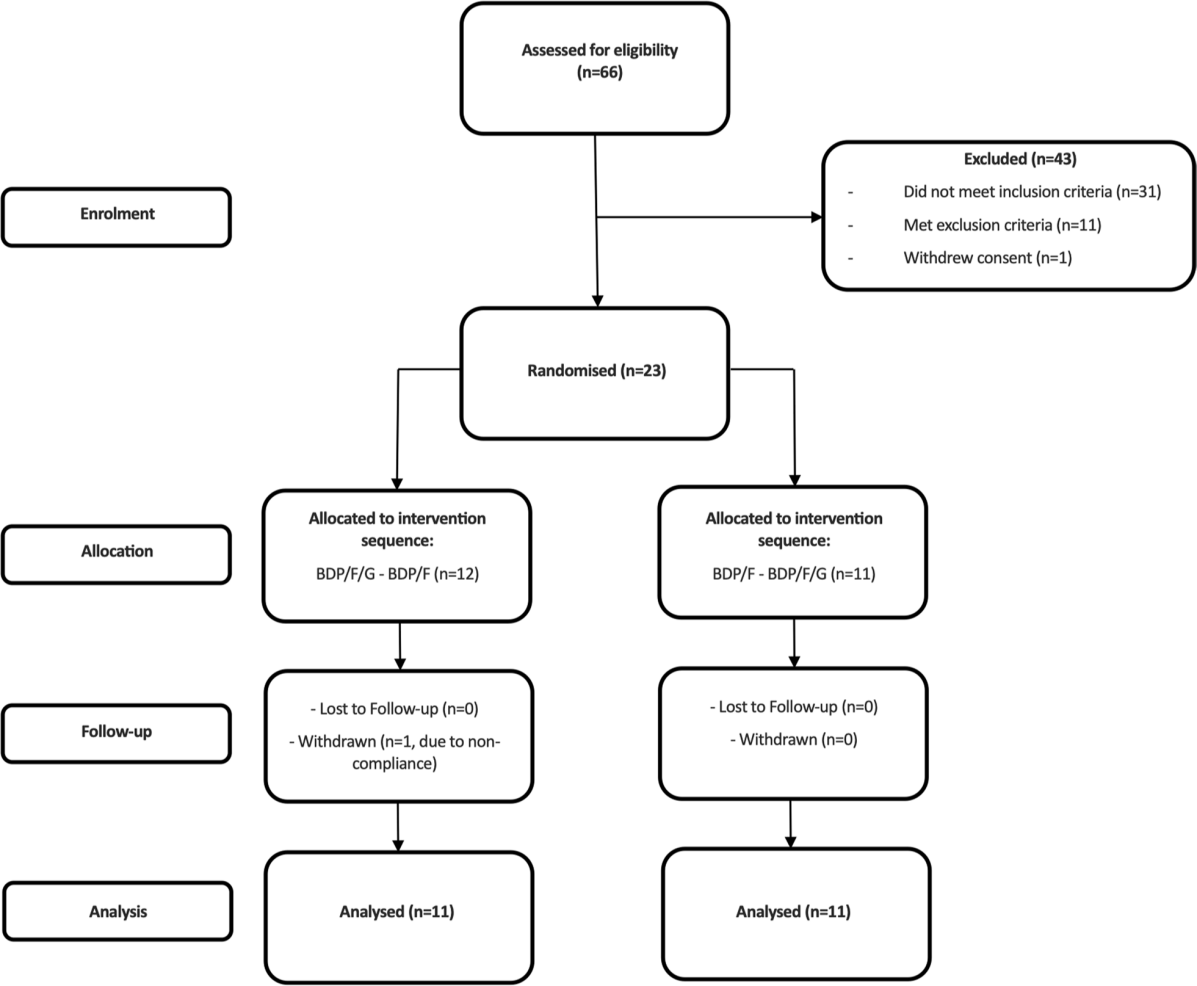
Patient flow through the study. *BDP* beclometasone dipropionate, *F* formoterol, *G* glycopyrronium

Sixty-six patients were screened, with 23 randomised, as shown in Fig. 2. One patient was withdrawn due to lack of treatment compliance, leaving 22 patients analysed. Patient characteristics are shown in Table 1. The mean FEV_1_% predicted (post-bronchodilator) was 49%, and RV was 152% predicted.

**Table 1:** Patient characteristics

Characteristic	n = 22 COPD
Gender, male/female	9/13
Age, years	64 (9)
Current/ex-smokers	9/13
Pack years	46 (25)
Prescribed dual^a^/triple^b^ inhaler therapy (n/n)	2/20
Prescribed supplemental oxygen (n)	0
Exacerbation history (n, zero/one/two exacerbation(s) in the last year)	13/8/1
CAT score	19.6 (8.4)
mMRC Dyspnoea Scale (n, grade 0/1/2/3/4)	3/10/4/4/1
FEV1 reversibility (%)	19 (13)
FEV_1_ (L)^c^	1.27 (0.34)
FEV_1_ (%)^c^	49 (9)
FVC (%)^c^	90 (10)
FEV_1_/FVC ratio (%)^c^	43 (11)
FEF25–75% (%)^c^	18 (7)
sGaw (L/s/kPa/L)	0.469 (0.195)
Raw (kPa/L/s)	0.512 (0.140)
RV (L)	3.16 (0.91)
RV (% predicted)	152 (35)
IC (% predicted)	80 (15)
FRC (% predicted)	139 (27)
TLC (% predicted)	114 (13)
R5 (kPa/L/s)^c^	0.570 (0.172)
R20 (kPa/L/s)^c^	0.395 (0.103)
R5–R20 (kPa/L/s)^c^	0.176 (0.118)
X5 (kPa/L/s)^c^	− 0.289 (0.150)
∆X5 (kPa/L/s)^c^	0.215 (0.194)
F*res* (1/s)^c^	23.40 (5.07)
AX (kPa/L)^c^	2.427 (1.739)

Data is mean (SD) where appropriate

^a^ICS/LABA

^b^ICS/LABA/LAMA

^c^Post-bronchodilator

### Primary endpoints

**Fig. 3:**
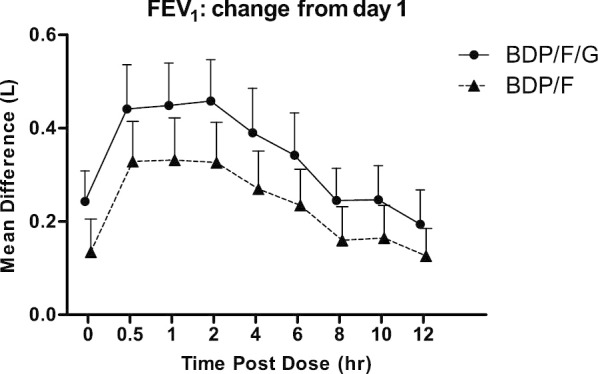
Mean FEV_1_ change on day 5 compared to Day 1 pre-dose value. Bars are 95% confidence intervals. *BDP* beclometasone dipropionate, *F* formoterol, *G* glycopyrronium

**Fig. 4:**
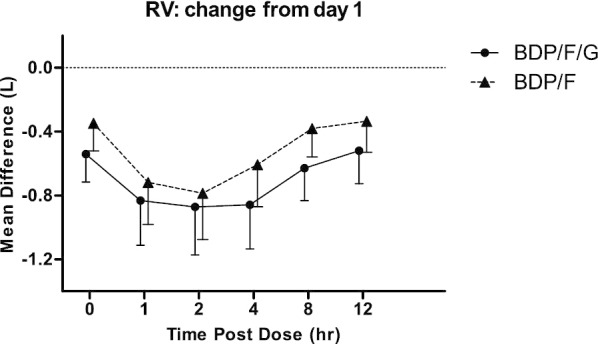
Mean residual volume (RV) change on day 5 compared to Day 1 pre-dose value. Bars are 95% confidence intervals. *BDP* beclometasone dipropionate, *F* formoterol, *G* glycopyrronium

Both BDP/F/G and BDP/F caused improvements in FEV_1_ and RV on day 5 compared to day 1 pre-dose (Figs. 3, 4). The FEV_1_ AUC_0–12_ change with BDP/F/G was greater compared to BDP/F; mean difference 104 mls; 95% CI 37, 171 mls; p = 0.0071. RV AUC_0–12_ change was greater with BDP/F/G compared to BDP/F; mean difference − 163 mls; 95% CI − 263, − 64 mls; p = 0.0028.

### Secondary endpoints

#### BDP/F/G versus BDP/F

BDP/F/G caused larger improvements than BDP/F for most of the lung function AUC_0–12_ parameters (Table 2). Using IOS, the change in small airway resistance, measured by R5–R20 AUC_0–12_, was greater with BDP/F/G compared to BDP/F (mean difference − 0.045 kPa/L/s; p = 0.0002). There were also significantly greater treatment differences in favour of BDP/F/G for Fres, AX and X5, while ∆X5 failed to reach statistical significance (p = 0.06). IC, TLC and FRC AUC_0–12_ measurements did not show statistically significant differences between treatments, although TLC and FRC showed numerical changes in favour of BDP/F/G. sGaw AUC_0–12_ change was greater with BDP/F/G (p = 0.01).

**Table 2:** AUC_0–12_ change from day 1

Parameter	BDP/F/G	BDP/F	BDP/F/G – BDP/F	
			Mean difference	p value
FVC (mls)	545 (414, 676)	339 (208, 470)	206 (54, 359)	0.0116
FEF25–75% (L/s)	0.110 (0.077, 0.143)	0.083 (0.050, 0.117)	0.026 (0.001, 0.051)	0.0395
sGaw (L/s/kPa/L)	0.311 (0.242, 0.381)	0.213 (0.143, 0.282)	0.099 (0.031, 0.167)	0.0101
Raw (kPa/L/s)	− 0.279 (− 0.323, − 0.236)	− 0.222 (− 0.266, − 0.179)	− 0.057 (− 0.077, − 0.037)	< 0.0001
IC (mls)	257 (135, 379)	260 (138, 382)	3 (− 146, 139)	0.96
FRC (mls)	− 490 (− 632, − 348)	− 387 (− 529, − 245)	− 103 (− 217, 11)	0.07
TLC (mls)	− 218 (− 319, − 117)	− 137 (− 238, − 36)	− 81 (− 196, 34)	0.16
R5–R20 (kPa/L/s)	− 0.163 (− 0.195, − 0.131)	− 0.118 (− 0.150, − 0.086)	− 0.045 (− 0.065, − 0.025)	0.0002
X5 (kPa/L/s)	0.203 (0.155, 0.252)	0.156 (0.108, 0.205)	0.047 (0.025, 0.069)	0.0003
∆X5 (kPa/L/s)	− 0.137 (− 0.219, − 0.056)	− 0.095 (− 0.177, − 0.014)	− 0.042 (− 0.086, 0.002)	0.06
F*res* (1/s)	− 7.201 (− 9.091, − 5.310)	− 5.005 (− 6.896, − 3.115)	− 2.195 (− 3.451, − 0.940)	0.0016
AX (kPa/L)	− 2.911 (− 3.467, − 2.356)	− 2.205 (− 2.761, − 1.650),	− 0.706 (− 1.047, − 0.365)	0.0004

Data = mean (95% CI) change in day 5 AUC_0–12_ from day 1. Treatment difference = BDP/F/G (AUC_0–12_ change at day 5 from day 1) – BDP/F (AUC_0–12_ change at day 5 from day 1)

Change in peak FEV_1_ was greater for BDP/F/G versus BDP/F (mean difference 120 mls; p = 0.0016), while for peak RV the difference was not significant (mean difference − 79 mls; p = 0.11). Other peak lung function results are shown in the Additional File 1; R5–R20 peak change with BDP/F/G was greater compared to BDP/F (mean difference − 0.036 kPa/L/s; p = 0.0022), while there were also significant differences for Fres, AX and X5, but not ∆X5. Lung volume peak measurements did not show statistically significant differences between treatments, while sGaw changes were in favour of BDP/F/G.

Trough RV improvement was greater for BDP/F/G compared with BDP/F, (− 179 mls; p = 0.0097), while the treatment difference for trough FEV_1_ (65 mls; p = 0.08) did not reach statistical significance (see Additional File 1).

#### BDP/F/G and BDP/F change from baseline

The baseline AUC_0–12_ measurements were performed while patients were taking BDP alone. The differences between baseline measurements and BDP/F/G or BDP/F treatments (day 5) are shown in Table 3. The comparisons of BDP/F with the baseline measurements showed differences in FEV_1_ AUC_0–12_ (mean difference 227 mls), RV AUC_0–12_ (mean difference − 558 mls) and R5–R20 AUC_0–12_ (mean difference − 0.117 kPa/L/s), all p < 0.0001. The comparisons of BDP/F/G with the baseline measurements showed differences in FEV_1_ AUC_0–12_ (mean difference 320 mls), RV AUC_0–12_ (mean difference − 678 mls) and R5–R20 AUC_0–12_ (mean difference − 0.165 kPa/L/s), all p < 0.0001.

**Table 3:** AUC_0–12_ change from baseline

Parameter	BDP/F/G		BDP/F	
	AUC_0–12_ mean change	p value	AUC_0–12_ mean change	p value
FEV_1_ (mls)	320 (257, 384)	< 0.0001	227 (163, 290)	< 0.0001
FVC (mls)	524 (420, 627)	< 0.0001	351 (248, 455)	< 0.0001
FEF25–75% (L/sec)	0.112 (0.080, 0.144)	< 0.0001	0.082 (0.050, 0.113)	< 0.0001
sGaw (L/s/kPa/L)	0.310 (0.259, 0.362)	< 0.0001	0.224 (0.172, 0.275)	< 0.0001
Raw (kPa/L/s)	− 0.298 (− 0.369, − 0.226)	< 0.0001	− 0.242 (− 0.313, − 0.170)	< 0.0001
RV (mls)	− 678 (− 847, − 509)	< 0.0001	− 558 (− 727, − 389)	< 0.0001
IC (mls)	328 (219, 438)	< 0.0001	289 (180, 399)	< 0.0001
FRC (mls)	− 468 (− 612, − 323)	< 0.0001	− 426 (− 571, − 282)	< 0.0001
TLC (mls)	− 136 (− 243, − 28)	0.0144	− 132 (− 239, − 24)	0.0174
R5–R20 (kPa/L/s)	− 0.165 (− 0.198, − 0.132)	< 0.0001	− 0.117 (− 0.150, − 0.084)	< 0.0001
X5 (kPa/L/s)	0.208 (0.169, 0.248)	< 0.0001	0.159 (0.120, 0.198)	< 0.0001
∆X5 (kPa/L/s)	− 0.207 (− 0.262, − 0.152)	< 0.0001	− 0.140 (− 0.195, − 0.085)	< 0.0001
F*res* (1/s)	− 8.235 (− 10.108, − 6.363)	< 0.0001	− 5.600 (− 7.472, − 3.727)	< 0.0001
AX (kPa/L)	− 2.991 (− 3.614, − 2.368)	< 0.0001	− 2.228 (− 2.851, − 1.604)	< 0.0001

Data = mean (95% CI) change in day 5 AUC_0– 12_ from pre-randomisation baseline visit AUC_0–12_. All AUC_0–12_ values (day 5 and baseline) are relative to the pre-dose value at baseline

### Safety

There were no serious AEs (adverse events) leading to withdrawal from the study or discontinuation of treatment. Further safety information is described in the Additional File 1.

## Discussion

This study focused on COPD patients with evidence of gas trapping, measured by RV. For the primary endpoint analysis (AUC_0–12_), BDP/F/G had greater effects compared to BDP/F on FEV_1_ and RV (mean treatment differences; 104 mls and − 163 mls respectively). This demonstrates that the G component of extrafine BDP/F/G reduced gas trapping in COPD patients. Furthermore, the greater improvement in R5–R20 AUC_0–12_ for BDP/F/G compared to BDP/F demonstrates that G also reduced small airway resistance. Overall, these results indicate that G had a beneficial effect on small airway physiology leading to improvements in gas trapping.

A secondary analysis, comparing BDP/F versus BDP, showed that F improved FEV_1_, RV and IOS measurements including R5–R20 AUC_0–12_. This demonstrates benefits of F on small airway physiology which are associated with decreases in gas trapping. The effect sizes observed showed that F treatment for 5 days caused 558 mls improvement in RV AUC_0–12_ and a 227 mls improvement in FEV_1_ AUC_0–12_. This comparison to baseline analysis also showed the extra benefit of the addition of G for 5 days, as BDP/F/G versus BDP treatment differences were 678 mls for RV AUC_0–12_ and 320 mls for FEV_1_ AUC_0–12_. Overall, these results indicate a greater effect of the addition of the first bronchodilator (BDP/F versus BDP) than the addition of the second bronchodilator (BDP/F/G versus BDP/F). This smaller effect of the second bronchodilator has been observed in many previous studies [5, 18, 19], and may be due to reaching near to the maximum improvement that may be achieved with these bronchodilator drug classes.

The primary and secondary outcomes, describing improvements in different components of lung mechanics, provide insights into the physiological effects of the long acting bronchodilators within BDP/F/G. Gas trapping is associated with an increased burden of symptoms in COPD [20, 21], and pharmacological interventions to reduce gas trapping can improve exercise performance [22, 23]. The primary endpoint analysis showed that using BDP/F/G can reduce gas trapping to a greater degree compared to BDP/F. This physiological effect may be extremely useful in clinical practice, in order to optimise the reduction of gas trapping and hence associated symptoms. The short treatment duration, and limited sample size of the current study means that we could not properly assess changes in symptoms or exercise capacity. Nevertheless, the TRILOGY phase 3 study, conducted over one year, provided evidence of greater improvements at many of the time-points for symptoms and quality of life for treatment with BDP/F/G compared to BDP/F [10].

The peak lung function changes on day 5 (for the comparison of BDP/F/G versus BDP/F) followed the same pattern of results as AUC_0–12_, except RV showed a lower numerical difference (65 mls) that was not statistically significant (p = 0.08). This might be due to the reduction in gas trapping by the first bronchodilator (F) being relatively large at peak, leaving little room for improvement by the addition of the second bronchodilator (G). For both the AUC_0–12_ and peak measurements, RV had the greatest sensitivity out of all the lung volume measurements to measure differences between BDP/F/G versus BDP/F; this is likely related to the enrichment of the study population for individuals with RV > 120% predicted at screening.

Using IOS allowed measurements of airway resistance and reactance to be collected. R5–R20 is a well accepted measurement of small airway resistance, and previous papers have shown that bronchodilators improve R5–R20 in COPD patients [4, 24]. In this study, the concurrent improvements in R5–R20 and RV imply that bronchodilator related improvements in small airway function enabled reduced gas trapping. IOS reactance measurements (X5, Fres, AX) reflect lung compliance and elasticity, and here we observed consistent improvements in these parameters for the G and F components of extrafine triple therapy.

Small airway closure can prevent low frequency oscillometric signals from reaching the distal lung [7]. Small airway closure can therefore operate as regional “choke points” during expiration but not inspiration, which has been called expiratory flow limitation (EFL). This can cause marked differences in reactance measurements during tidal breathing, and is measured by ∆X5, which is an oscillometry measurement of EFL. It has been reported that EFL measurements are associated with greater airflow limitation and gas trapping, greater symptoms, more exacerbations and increased mortality [21, 25, 26]. The comparisons of BDP/F/G or BDP/F versus BDP (baseline) both showed large improvements in ∆X5, and consequently the BDP/F/G versus BDP/F comparison yielded a small treatment difference that was not statistically significant.

The design of the study, using the same inhaler device delivering extrafine formulations for all treatments, allowed the contributions of F and G on small airway function and gas trapping to be determined. We did not study a non-extrafine formulation with less peripheral lung deposition compared to BDP/F/G; this would be an interesting future study to compare changes in small airway function and gas trapping between treatments. While this was an open study, the objective nature of the endpoints (lung function measurements) restricted any bias that could be introduced by lack of treatment blinding. The main advantage of blinding is for patient reported outcomes which are more subjective. A potential limitation of the study was the relatively small sample size. However, the sample size was very similar to other crossover design studies investigating long acting bronchodilator effects on lung function [27, 28], and the enrichment for individuals with gas trapping (RV > 120% predicted) increased the homogeneity of the population thereby decreasing the potential variability in lung function data.

The mean CAT score at screening was 19.6, suggesting this was a very symptomatic population. However, 13 out of the 22 patients had mMRC scores < 2 at study entry. The majority of patients (20 out of 22) were already receiving triple therapy in real life at study entry, which likely reduced the burden of dyspnoea. The differences between CAT and mMRC scores likely reflect the broader range of disease components assessed by the former, as previously reported [29]. All patients were using ICS at study entry; these drugs should be used in patients with a history of exacerbations [30], but often are inappropriately given to patients who do not have a history of exacerbations. While some of our study population had no exacerbations in the last year, this may reflect a positive treatment response to ICS initiation [31].

Late phase studies with large sample sizes often require statistical approaches to account for multiple testing. This smaller study was more exploratory in nature, providing information on the usefulness of a broad range of lung function tests. This information can be used to select the most appropriate tests to be used in future studies comparing inhaled treatments. Multiple testing correction is often not performed in smaller clinical trials with a more exploratory nature [32]. Nevertheless, many of the p values reported were highly significant, arguing against the presence of false positives due to multiple testing.

In summary, this study focused on COPD patients with gas trapping, and demonstrated that the G and F components of extrafine BDP/F/G improved FEV_1_, RV and small airway function. It can be concluded that these long acting bronchodilators target small airway function, thereby improving gas trapping and airflow.

## Supplementary Information

**Additional file 1**. Triflow Supplement.

## Data Availability

The datasets used and/or analysed during the current study are available from the corresponding author on reasonable request.

## Abbreviations

BDP: Beclometasone dipropionate
F: Formoterol
G: Glycopyrronium
COPD: Chronic obstructive pulmonary disease
ICS: Inhaled corticosteroid
LABA: Long acting beta-agonist
LAMA: Long acting muscarinic antagonist
ECG: Electrocardiogram
pMDI: Pressurised metered dose inhaler
IOS: Impulse oscillometry
CAT: COPD assessment test
mMRC: Modified medical research council
FEV_1_: Forced expired volume in 1 s
AUC_0–12_: Area under the curve over 12 h
FVC: Forced vital capacity
FEF25–75%: Forced expiratory flow between 25 and 75% of FVC
sGaw: Specific airway conductance
Raw: Airway resistance
RV: Residual volume
IC: Inspiratory capacity
FRC: Functional residual capacity
TLC: Total lung capacity
R5: Total respiratory resistance
R20: Central respiratory resistance
R5–R20: Peripheral respiratory resistance
X5: Respiratory reactance
∆X5: Expiratory flow limitation
F*res*: Resonance frequency
AX: Reactance area
CI: Confidence interval
AEs: Adverse events
EFL: Expiratory flow limitation
