# Supplementary material for: The TRIFLOW study: a randomised, cross-over study evaluating the effects of extrafine beclometasone/formoterol/glycopyrronium on gas trapping in COPD
Source: Respir Res. 2020 Dec 9;21:323. doi: 10.1186/s12931-020-01589-5 (PMC7727250; doi:10.1186/s12931-020-01589-5)
Supplement: Supplementary file 1 — Additional file 1. Triflow Supplement. [file 12931_2020_1589_MOESM1_ESM.docx]

**Triflow Supplement**

**Secondary Endpoints**

**Table S1.** Peak change from day 1

| Parameter | BDP/F/G | BDP/F | Treatment difference | |
| --- | --- | --- | --- | --- |
|  |  |  | Mean difference | p value |
| **FEV**_1_  **(mls)** | 503  (407, 600) | 384  (288, 480) | 120  (55, 184) | 0.0016 |
| **FVC**  **(mls)** | 850  (658, 1042) | 607  (415, 798) | 243  (68, 419) | 0.0106 |
| **FEF25-75% (L/sec)** | 0.210  (0.155, 0.266) | 0.166  (0.110, 0.222) | 0.044  (0.001, 0.088) | 0.0442 |
| **sGaw (L/s/kPa/L)** | 0.466  (0.367, 0.564) | 0.365  (0.266, 0.463) | 0.101  (0.009, 0.193) | 0.0335 |
| **Raw (kPa/L/s)** | -0.351  (-0.387, -0.315) | -0.309  (-0.345, -0.273) | -0.042  (-0.069, -0.015) | 0.0045 |
| **RV**  **(mls)** | -947  (-1129, -766) | -869  (-1050, -688) | -79  (-176, 19) | 0.11 |
| **IC**  **(mls)** | 458  (293, 622) | 470  (305, 634) | -12  (-169, 145) | 0.87 |
| **FRC**  **(mls)** | -713  (-892, -533) | -660  (-840, -480) | -53  (-156, 50) | 0.29 |
| **TLC**  **(mls)** | -391  (-495, -287) | -306  (-410, -202) | -85  (-184, 15) | 0.09 |
| **R5-R20 (kPa/L/s)** | -0.221  (-0.254, -0.188) | -0.185  (-0.218, -0.152) | -0.036  (-0.057, -0.015) | 0.0022 |
| **X5**  **(kPa/L/s)** | 0.266  (0.225, 0.308) | 0.231  (0.190, 0.272) | 0.036  (0.003, 0.068) | 0.0355 |
| **∆X5 (kPa/L/s)** | -0.240  (-0.300, -0.181) | -0.210  (-0.270, -0.151) | -0.030  (-0.088, 0.028) | 0.29 |
| **F*res***  **(1/s)** | -11.130  (-13.386, -8.874) | -8.613  (-10.869, -6.356) | -2.517  (-3.830, -1.205) | 0.0007 |
| **AX**  **(kPa/L)** | -3.770  (-4.266, -3.274) | -3.228  (-3.724, -2.732) | -0.542  (-0.918, -0.166) | 0.0072 |

**Abbreviations:**FEV_1_, forced expired volume in 1 second; FVC, forced vital capacity; FEF25-75%, forced expiratory flow between 25-75% of FVC; sGaw, specific airway conductance; Raw, airway resistance; RV, residual volume; IC, inspiratory capacity; FRC, functional residual capacity; TLC, total lung capacity; R5-R20, peripheral respiratory resistance; X5, respiratory reactance; ∆X5, expiratory flow limitation; F*res,* resonance frequency; AX, reactance area.

**Notes:** Data = mean (95% CI) change in post dose day 5peak from day 1. Treatment difference = BDP/F/G (change in peak value at post dose day 5 from day 1) – BDP/F (change in peak value at post dose day 5 from day 1).

**Table S2.** Trough change from day 1

| Parameter | BDP/F/G | BDP/F | Treatment difference | |
| --- | --- | --- | --- | --- |
|  |  |  | Mean difference | p value |
| **FEV_1_**  **(mls)** | 175  (115, 234) | 109  (50, 168) | 65  (-9, 139) | 0.08 |
| **RV**  **(mls)** | -446  (-601, -292) | -268  (-422, -113) | -179  (-308, -49) | 0.0097 |

**Abbreviations:**FEV_1_, forced expired volume in 1 second; RV, residual volume.

**Notes:** Data = mean (95% CI) change in post dose day 5troughfrom day 1. Treatment difference = BDP/F/G (change in trough value at post dose day 5 from day 1) – BDP/F (change in trough value at post dose day 5 from day 1).

**Baseline**

**Table S3.** Peak change from baseline visit

| Parameter | BDP/F/G | | BDP/F | |
| --- | --- | --- | --- | --- |
|  | Mean peak change | p value | Mean peak change | p value |
| FEV_1_  (mls) | 402  (326, 478) | <0.0001 | 293  (217, 369) | <0.0001 |
| RV  (mls) | -705  (-915, -495) | <0.0001 | -662  (-873, -452) | <0.0001 |

**Abbreviations:**FEV_1_, forced expired volume in 1 second; RV, residual volume.

**Notes:** Data = mean (95% CI) change in day 5peak valuefrom pre-randomisation baseline visitpeak value.

**Safety**

3 patients experienced at least 1 treatment emergent AE during BDP/F/G treatment and 7 patients during BDP/F treatment. All AEs were mild in severity except 1 (moderate dyspnoea during BDP/F treatment), and none were considered related to study treatment. There was a higher incidence of dyspnoea during the BDP/F treatment (n=4) versus BDP/F/G (n=0).Other adverse events included worsening COPD (mild, worsening on exertion), nasal congestion, and papilloma excision during BDP/F/G treatment, and dizziness, tension headache, ear discomfort and nausea during BDP/F treatment. Laboratory safety, vital signs, 12‑lead ECG and physical examination data were not analysed.
